# Forest defoliation by an invasive outbreak insect: Catastrophic consequences for a charismatic mega moth

**DOI:** 10.1002/ece3.70046

**Published:** 2024-08-19

**Authors:** Richard L. Lindroth, Mark R. Zierden, Clay J. Morrow, Patricia C. Fernandez

**Affiliations:** ^1^ Department of Entomology University of Wisconsin‐Madison Madison Wisconsin USA; ^2^ Department of Forest and Wildlife Ecology University of Wisconsin‐Madison Madison Wisconsin USA; ^3^ CONICET‐Universidad de Buenos Aires, Centro de Investigación de Hidratos de Carbono (CIHIDECAR), Ciudad Universitaria – Pabellón 2 Buenos Aires Argentina; ^4^ Departamento de Biología Aplicada y Alimentos, Cátedra de Química de Biomoléculas Universidad de Buenos Aires, Facultad de Agronomía Buenos Aires Argentina; ^5^ Present address: Department of Chemistry Lake Superior State University Sault Sainte Marie Michigan USA; ^6^ Present address: Forest Products Laboratory United States Forest Service Madison Wisconsin USA

**Keywords:** biodiversity, genetic variation, indirect ecological effects, induced defense, invasive species, phytochemical landscape

## Abstract

Earth is now experiencing declines in insect abundance and diversity unparalleled in human history. The drivers underlying those declines are many, complex, and incompletely known. Here, using a natural experiment, we report the first test of the hypothesis that forest defoliation by an invasive outbreak insect compromises the fitness of a native insect via damage‐induced increases in toxicity of the forest canopy. We demonstrate that defoliation by the invasive spongy moth (*Lymantria dispar*) elicits an average 8.4‐fold increase in foliar defense expression among aspen (*Populus tremuloides*) genotypes. In turn, elevated defense dramatically reduces survivorship, feeding, and growth of a charismatic mega moth (*Anthereae polyphemus*). This work suggests that changes to the phytochemical landscape of forests, mediated by invasive outbreak insects, are likely to negatively impact native insects, with potential repercussions for community diversity and ecosystem function across expansive scales.

## Graphical abstract

The drivers underlying worldwide declines in insect abundance and diversity are multiple, complex, and incompletely known. We use a natural experiment to test the hypothesis that forest defoliation by an invasive outbreak insect compromises the fitness of a native insect via damage‐induced increases in toxicity of the forest canopy. We demonstrate that defoliation (I, II) by the invasive spongy moth (*Lymantria dispar*) elicits a dramatic increase in foliar defense expression (III, IV) by aspen (*Populus tremuloides*), which in turn reduces survivorship, feeding, and growth (V) of the charismatic polyphemus silk moth (*Anthereae polyphemus*).

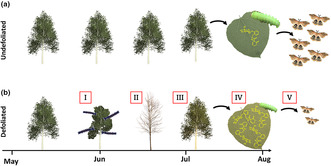

## 1 INTRODUCTION

Understanding the complex of anthropogenic factors driving the global decline of insect abundance and biodiversity has emerged as a major scientific challenge of the 21st century (Rumohr et al., 2023; Sánchez‐Bayo & Wyckhuys, 2019; Wagner, 2020; Wagner et al., 2021). Time series of insect abundance data collected over long periods have been instrumental in documenting declines and have implicated multiple drivers, contributing to the notion of “death by a thousand cuts” (Wagner et al., 2021). Long‐term observational studies have been less useful, however, in identifying specific drivers of decline, the impacts of which vary across time, space, and species. For that purpose, experiments are critically needed (Weisser et al., 2023).

**FIGURE 1:**
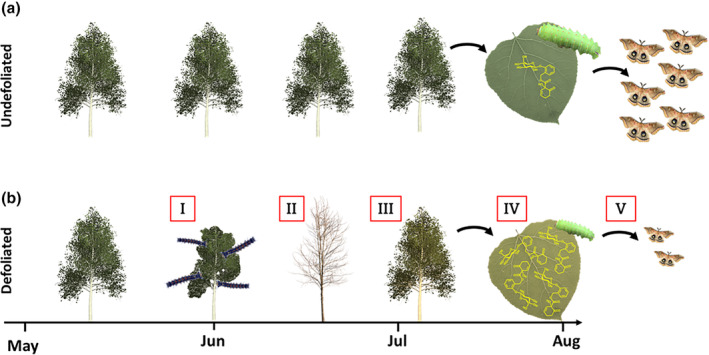
Conceptualization of indirect, chemically‐mediated, legacy effects of defoliation by an invasive insect species on fitness of a native species. (a) and (b) represent forests undefoliated and defoliated, respectively, by spongy moth (*Lymantria dispar*). (I) Defoliation of aspen by outbreak populations of spongy moths accelerates in June, leading to nearly complete destruction (II) of the foliar canopy. (III) Aspen trees refoliate, with leaves containing high concentrations of salicinoid defense compounds (IV), relative to undefoliated trees. (V) Polyphemus silk moth caterpillars feeding on refoliated trees experience reduced survivorship and growth, relative to caterpillars feeding on undamaged trees.

Introduced invasive species are regarded among the most important drivers of insect declines (Sánchez‐Bayo & Wyckhuys, 2019; Wagner et al., 2021), yet knowledge of the specific ecological mechanisms by which such impacts occur is poor. Invasive forest insects are of particular concern, as they can profoundly alter the diversity, structure, and function of forest ecosystems across enormous regions (Boyd et al., 2013; Gandhi & Herms, 2010; Kenis et al., 2009; Weisser et al., 2023). Population outbreaks can defoliate forests, directly harming co‐occurring insects via depletion of food resources (Timms & Smith, 2011; Work & McCullough, 2000). Alternatively, invasive outbreak insects may affect native insects indirectly, through induction of chemical defenses in damaged trees (Figure 1). Such responses could extend the detrimental consequences of defoliation to other insect species long past the period of active feeding. Here, for the first time, we report that defoliation by an invasive forest insect species dramatically increases the toxicity of the forest canopy, thereby reducing fitness of a charismatic native species. For invasive defoliators and host trees with broad range distributions, such indirect, phytochemically‐mediated impacts on native fauna have the potential to propagate over expansive forest landscapes.

### 1.1 The ecological players

**FIGURE 2:**
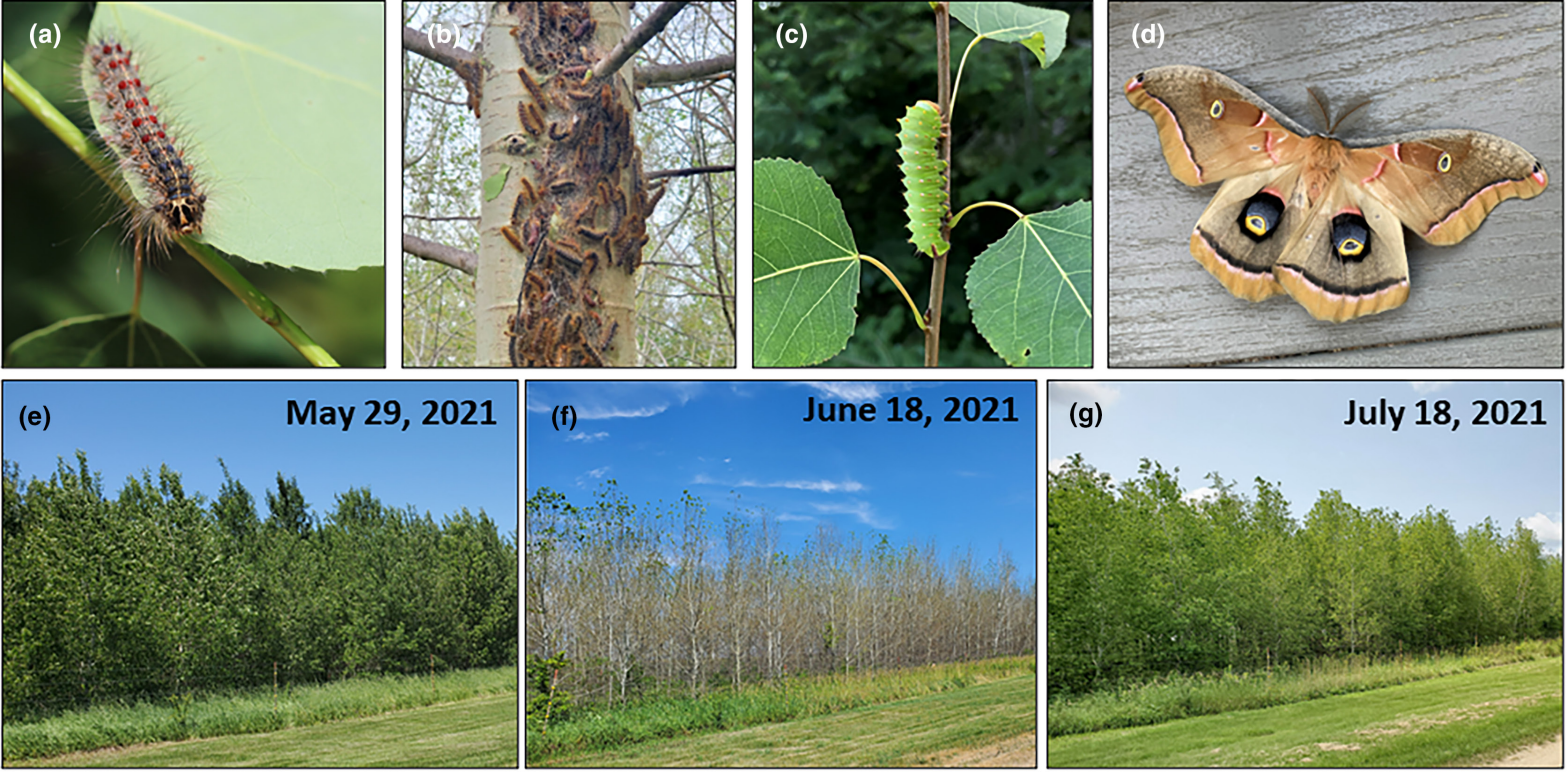
The ecological players. (a) Larval spongy moth (*Lymantria dispar*). (b) Aggregation of spongy moth larvae on aspen tree. (c) Larval polyphemus silk moth (*Anthereae polyphemus*). (d) Adult polyphemus silk moth. (e–g) Timeline of aspen (*Populus tremuloides*) forest defoliation and refoliation. Photo credits: a–d, R.L. Lindroth; e–g, M.R. Zierden.

The spongy moth (formerly “gypsy moth”; *Lymantria dispar*, Figure 2a,b) is the most damaging invasive defoliator of forests in North America (Mattson et al., 1991). Originally introduced to Massachusetts, it is now established in hardwood forests throughout much of the eastern and northcentral USA. Larvae emerge synchronous with tree budbreak and early leaf‐out (April–May), complete their feeding by early summer (June), and at outbreak densities can strip canopies bare (Doane & McManus, 1981; Stoyenoff et al., 1994; Sui et al., 2023) (Figure 2e–g).

**FIGURE 3:**
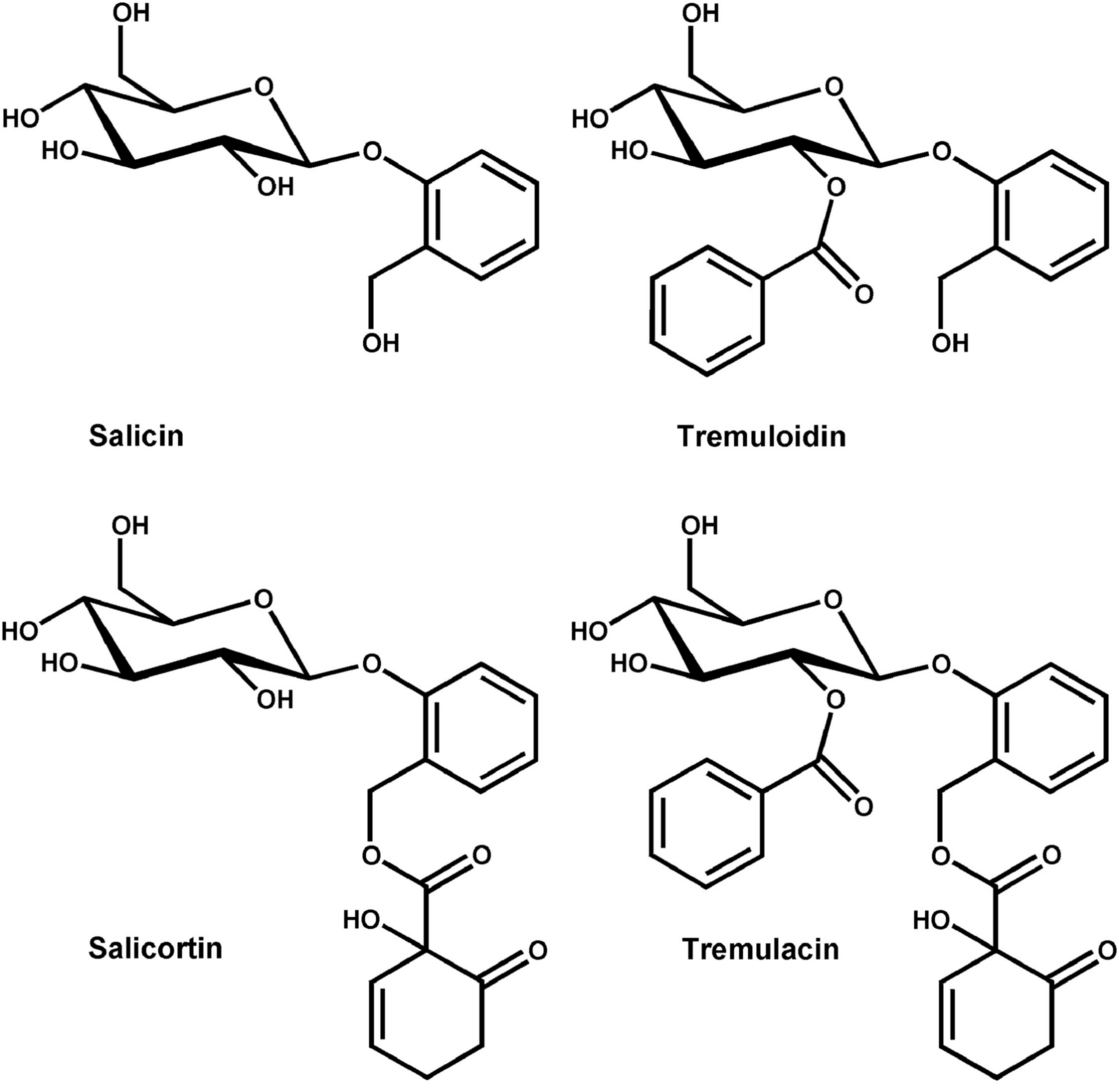
Principal salicinoid phenolic glycosides (SPGs) in trembling aspen. Salicortin and tremulacin contain a 1‐hydroxy‐6‐oxocyclohex‐2‐ene‐1‐carboxylic acid (6‐HCH) functional group, and typically constitute >95% of the total SPG pool.

Trembling aspen (*Populus tremuloides*) is the most widespread tree species in North America (Perala, 1990). It is a foundation species in 56 north‐temperate, boreal, and montane forests, and frequently exists as nearly mono‐specific stands of mixed genotypes (Long & Mock, 2012; Mitton & Grant, 1996; Perala, 1990). Aspen exhibits extraordinary genotypic variation, including expression of its signature chemical defense compounds, salicinoid phenolic glycosides (SPGs; Figure 3) (Cole et al., 2021; Lindroth & St. Clair, 2013). Despite its chemical armament, however, aspen is a favored hostplant of many insect species, including spongy moth in the Great Lakes Region. Following major defoliation events, aspen trees refoliate within several weeks (Figure 2e–g) but exhibit reduced growth for the year (Duncan & Hodson, 1958; Sui et al., 2023).

The charismatic native silk moth, *Anthereae polyphemus*, is the second largest moth species in North America, broadly distributed in hardwood forests (Tuskes et al., 1996) (Figure 2c,d). Polyphemus larvae are adapted to feed on numerous tree species, including aspen, in mid‐ to late‐summer. In years with irruptive spongy moth outbreaks, the caterpillars are forced to feed on reflushed tree foliage.

### 1.2 A natural experiment

In 2021, a localized spongy moth outbreak in southern Wisconsin, USA, provided a natural experiment by which to test the hypothesis that defoliation induces production of chemical defenses that, in turn, harm native insects. We used two experimental aspen forests, located 6 km apart, that incorporated a common set of multiple aspen genotypes of similar age. One forest (hereafter, “Defoliated”) was heavily infested with spongy moth egg masses in spring, 2021. The second forest (hereafter, “Undefoliated”) contained none. Eggs hatched in early May, simultaneous with leaf flush and expansion. Canopy defoliation approached 100% by the time larvae pupated in late June. When aspen experience heavy defoliation, they respond by shedding petioles and leaf fragments, and reflushing a new set of leaves from axillary buds (Donaldson & Lindroth, 2008; Osier & Lindroth, 2001). By early‐mid July, our defoliated aspen trees produced a second flush of leaves (Figure 2e–g). To investigate how spongy moth defoliation may alter chemical defense expression, we monitored levels of SPGs in aspen tree foliage. Next, to explore potential chemical legacy effects of defoliation on a native silk moth species, we conducted feeding bioassays with polyphemus caterpillars.

## 2 METHODS

### 2.1 Experimental forests

The experimental aspen forests used in this research were planted in 2010 at the University of Wisconsin's Arlington Agricultural Research Station (43.3° N latitude, 89.3° W longitude). The forests were established on former grass/hay fields (silt loam soil) near adjoining, established woodlots, and were separated by 6 km of crop and pasture land. At the time of this research (2021), the forests had closed canopies and the trees were reproductively mature.

Our Defoliated forest contained 510 aspen genotypes (2–6 replicates each) collected from a north–south gradient through Wisconsin, USA. The Undefoliated forest contained a subset of 14 of those genotypes, collected from south‐central Wisconsin (~45 replicates each). Trees were genotyped by sequencing or microsatellite analysis and showed no evidence of population structure (Barker, Riehl, et al., 2019).

Detailed information about establishment of the Defoliated forest is provided by Barker, Riehl, et al. (2019) and for the Undefoliated forest by Cope, Keefover‐Ring, et al. (2021). Numerous tree functional traits, particularly growth and phytochemistry, were monitored for both forests during the period 2014–2019 (Barker et al., 2018; Barker, Riehl, et al., 2019; Cole et al., 2021; Cope, Keefover‐Ring, et al., 2021; Cope, Lindroth, et al., 2021).

### 2.2 Aspen phytochemistry

For phytochemical analyses, leaves were collected from three trees of each of eight genotypes in the Defoliated forest in July 2020 and monthly from May through July 2021. Similar collections, from the same genotypes, were made in the Undefoliated forest as part of another research project in July 2020. Samples consisted of 16 leaves randomly harvested from four separate branches on each tree, using pole pruners. The samples were stored on ice for transport to our laboratory, where they were vacuum dried, pulverized in a ball mill, and stored at −20°C until analysis.

The aspen foliar constituents most likely to influence insect performance are protein (typically measured as total N) and two forms of secondary metabolites: salicinoid phenolic glycosides (SPGs) and condensed tannins (CTs) (Barker, Holeski, & Lindroth, 2019; Lindroth & St. Clair, 2013). Foliar nitrogen concentrations were determined using combustion gas chromatography (Flash EA 1112, Thermo Finnigan, Milan, Italy). Salicinoid phenolic glycosides were extracted into methanol, separated by reverse phase UPLC, and quantified using electrospray ionization single quadrupole mass spectrometry (Acuity iClass UPLC/MS system, Waters, Milford, MA, USA) in negative ion mode following the method of Rubert‐Nason et al. (2018). We calculated total SPGs as the sum of salicin, salicortin, tremulacin and tremuloidin; salicortin and tremulacin constituted >95% of the total values. Condensed tannins were extracted into 70% acetone with 10 mM ascorbic acid and quantified via the acid butanol method (Porter et al., 1986). Condensed tannin standards were purified from aspen foliage by adsorption chromatography (Hagerman & Butler, 1980).

### 2.3 Polyphemus bioassays

**FIGURE 4:**
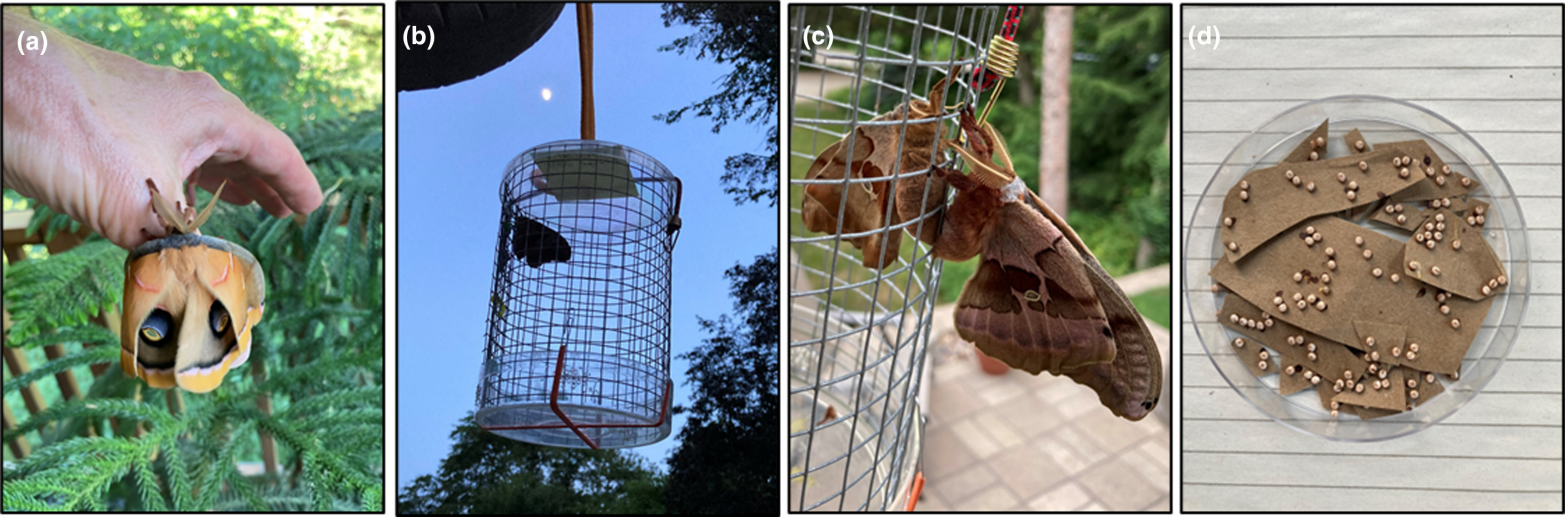
Protocol for polyphemus breeding. (a) Newly eclosed polyphemus moth, wings not yet fully expanded. (b) Caged female moth placed outside at night. (c) Copulating female (left) and male (right) moths. (d) Eggs. Photo credits: R.L. Lindroth.

This work was conducted to assess the response of a native, summer‐feeding saturniid species, *Antheraea polyphemus*, to defoliation‐mediated changes in the quality of aspen foliage. Polyphemus cocoons were obtained from Magic Wings Butterfly Conservatory (Deerfield, MA, USA) from a local population. Upon eclosion, females were placed outside in hardware cloth cages and allowed to mate with local (Wisconsin) male polyphemus moths (Figure 4). Resulting eggs provided larvae for use in feeding bioassays. Upon hatching, neonate larvae were placed into ventilated, 2.5 × 15 cm plastic dishes and fed non‐experimental oak and/or aspen foliage. The larvae were maintained in a Percival growth chamber under a 24:18°C temperature regimen and 15:9 h light:dark cycle.

**FIGURE 5:**
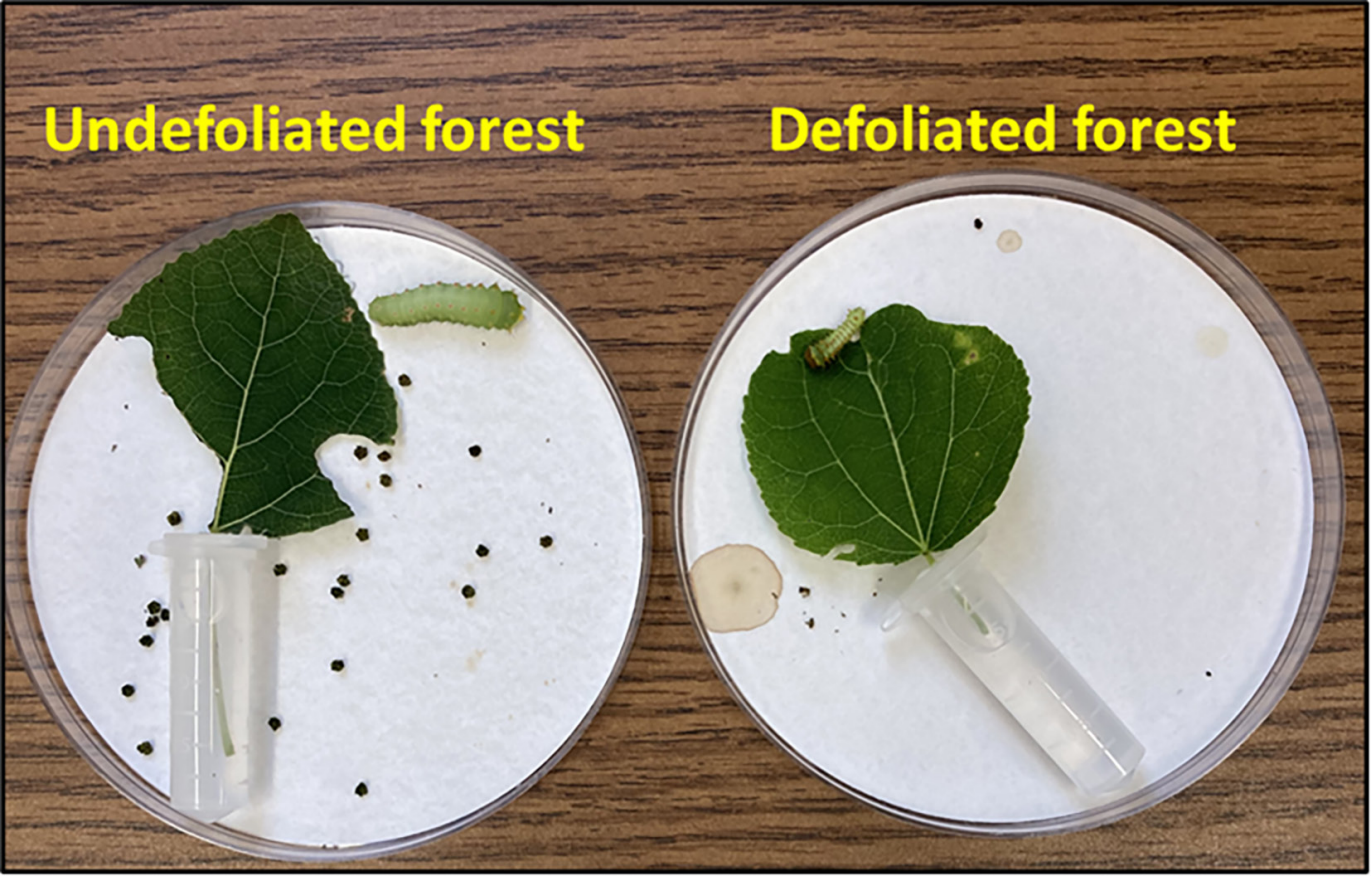
Bioassay set‐up. Note considerable feeding, frass production and larval size for polyphemus fed undamaged leaves from the Undefoliated forest versus refoliated leaves from the Defoliated forest. Shrunken appearance and brown stains (from regurgitant) are signs of distress in the caterpillar fed refoliated leaves. Photo credit: R.L. Lindroth.

We conducted feeding bioassays with third stadium caterpillars to determine host plant effects on several performance variables linked to insect fitness, including survivorship, growth, and food consumption. The use of such metrics as proxies for fitness is well‐established in insect ecology (Awmack & Leather, 2002; Lampasona et al., 2022; Scriber & Slansky Jr., 1981). Recently molted third instars (without access to foliage) were selected, weighed, and placed individually into plastic 1 × 10 cm rearing dishes containing a weighed aspen leaf from an experimental treatment (Figure 5). We conducted two bioassays for each experimental tree, with four trees per genotype, and a common set of five genotypes for the Defoliated and Undefoliated forests (total of 80 insect bioassays). Larvae from five different mothers were distributed across, rather than within, treatments to reduce the potential for confounding maternal effects. Larvae were maintained for the duration of the third stadium in a Percival growth chamber at 24:18°C and 15:9 h light: dark cycle. A subset of ten newly molted third instars were frozen and lyophilized for determining wet: dry weight ratios. We conducted the bioassays under standardized conditions, as opposed to in situ on trees, to focus on how defoliation‐mediated effects on foliar quality per se influence herbivore performance, independent of the effects of defoliation on environmental conditions (e.g., temperature, humidity). This bioassay approach has been used extensively in previous aspen‐insect studies (Hemming & Lindroth, 1995; Hwang & Lindroth, 1997; Osier & Lindroth, 2006).

Experimental foliage was collected between 0900 and 1300 h, in the same manner as described for chemical analyses. A subset of leaves from each field collection was weighed, frozen and lyophilized to determine wet: dry weight ratios. Another subset was processed for phytochemistry, as described previously. Bioassay leaves were kept hydrated by inserting the petioles into 6 mL florist water piks filled with water. All rearing dishes were examined 3 times per day and additional leaves were provided if more than 90% of the existing leaf material had been consumed. Uneaten leaves from each rearing dish were collected daily and stored frozen. Upon completion of the molt into the fourth stadium, individual larvae were frozen. When all bioassays had been completed, larvae and leaf remnants were lyophilized and weighed. Those data, along with corresponding wet: dry weight ratios for leaves and larvae, were used to calculate larval relative growth rate (RGR) (biomass gained ⸳ initial larval biomass^−1^ ⸳ day^−1^) and relative consumption rate (RCR) (food ingested ⸳ initial larval biomass^−1^ ⸳ day^−1^) (Waldbauer, 1968).

### 2.4 Statistics

Preliminary evaluation of our data revealed no association between foliar N or condensed tannin (CT) concentrations and polyphemus performance. Those phytochemical traits were therefore omitted from this report, to facilitate a focus on the highly influential salicinoid phenolic glycosides.

We evaluated the effects of spongy moth defoliation on the Defoliated forest by comparing SPG concentrations among eight genotypes across multiple time points. A repeated measures analysis of variance (ANOVA) was conducted to assess variation contributed by genotype, time, and genotype × time interaction. We also conducted Tukey's pairwise *t*‐tests to compare SPG concentrations among individual time points. We used multifactor regression ANOVA to explore the effects of defoliation, genotype, and their interaction on post‐outbreak SPG concentrations, and polyphemus growth and consumption rates. Defoliation was treated as a categorical variable because we compared two classes of leaves: first flush (not defoliated) vs. reflushed (second flush, following defoliation). All ANOVAs for the bioassay component of this study were conducted on nested models to account for the non‐independence of replicate trees within genotypes. To test the effects of spongy moth defoliation on polyphemus survivorship, we conducted an Analysis of Deviance comparing logistic models of increasing complexity and quantifying the additional variation explained at each step. We assessed the effects of defoliation, genotype, SPG concentrations, and all interactions, on larval survivorship. The SPG LC_50_ (concentration lethal to 50% of bioassay larvae) was calculated by fitting a logistic regression of survivorship against SPG concentration. LC_50_ is equivalent to the negative of the intercept divided by the coefficient for SPGs from the fitted survivorship model. SPG concentrations across the Undefoliated and Defoliated forests were compared with each other and with the LC_50_ value using Tukey's pairwise *t*‐tests. We compared models of polyphemus fitness metrics (survivorship, RGR, and RCR) with and without SPGs as an independent variable to evaluate the degree to which Genotype and Forest effects are explained by SPG concentrations. Finally, to evaluate whether the two experimental forests differed in SPG expression prior to the spongy moth outbreak, we conducted an ANOVA comparing SPG concentrations between forests and among genotypes in 2020. Adjusted *R* ^2^ values were calculated for all underlying models. All pairwise tests were conducted with least‐squares means (Lenth, 2023) among groups rather than with raw means. All assumptions were assessed and met for each statistical test. Statistical analyses were conducted using the R statistical software package (R Core Team, 2021).

## 3 RESULTS

### 3.1 Defoliation elevates expression of salicinoid defense compounds in aspen

**FIGURE 6:**
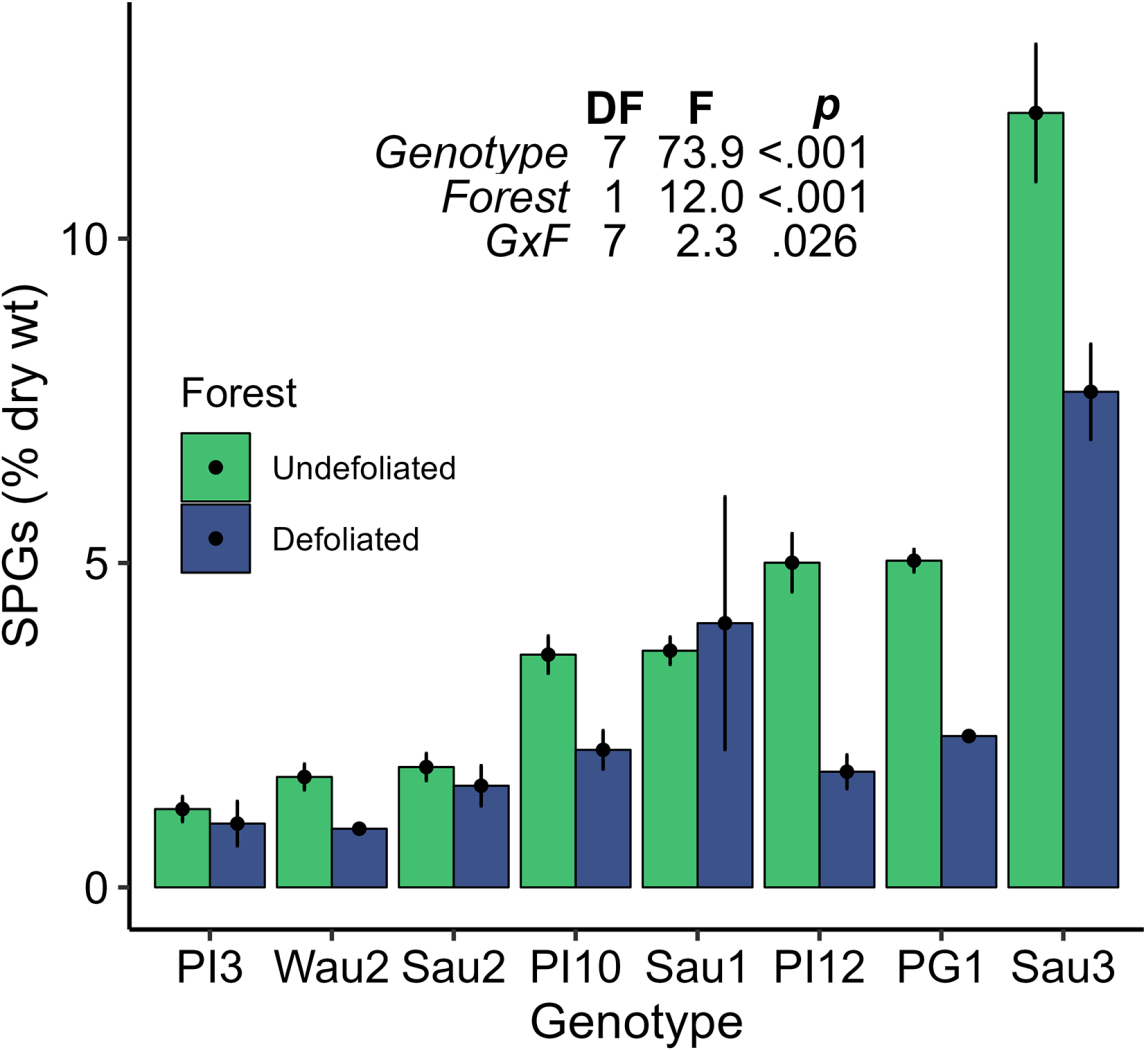
Comparison of salicinoid phenolic glycoside (SPG) levels between experimental forests in midsummer of the year (2020) preceding spongy moth defoliation. Bars indicate mean total SPGs, ± 1 standard error, for each genotype (8) within the two aspen forests. The embedded table shows the ANOVA degrees of freedom, F‐statistic, and *p*‐value for effects of genotype, forest, and their interaction. Among eight aspen genotypes, SPG levels were similar, or *lower*, in the Defoliated forest relative to the Undefoliated forest.

**FIGURE 7:**
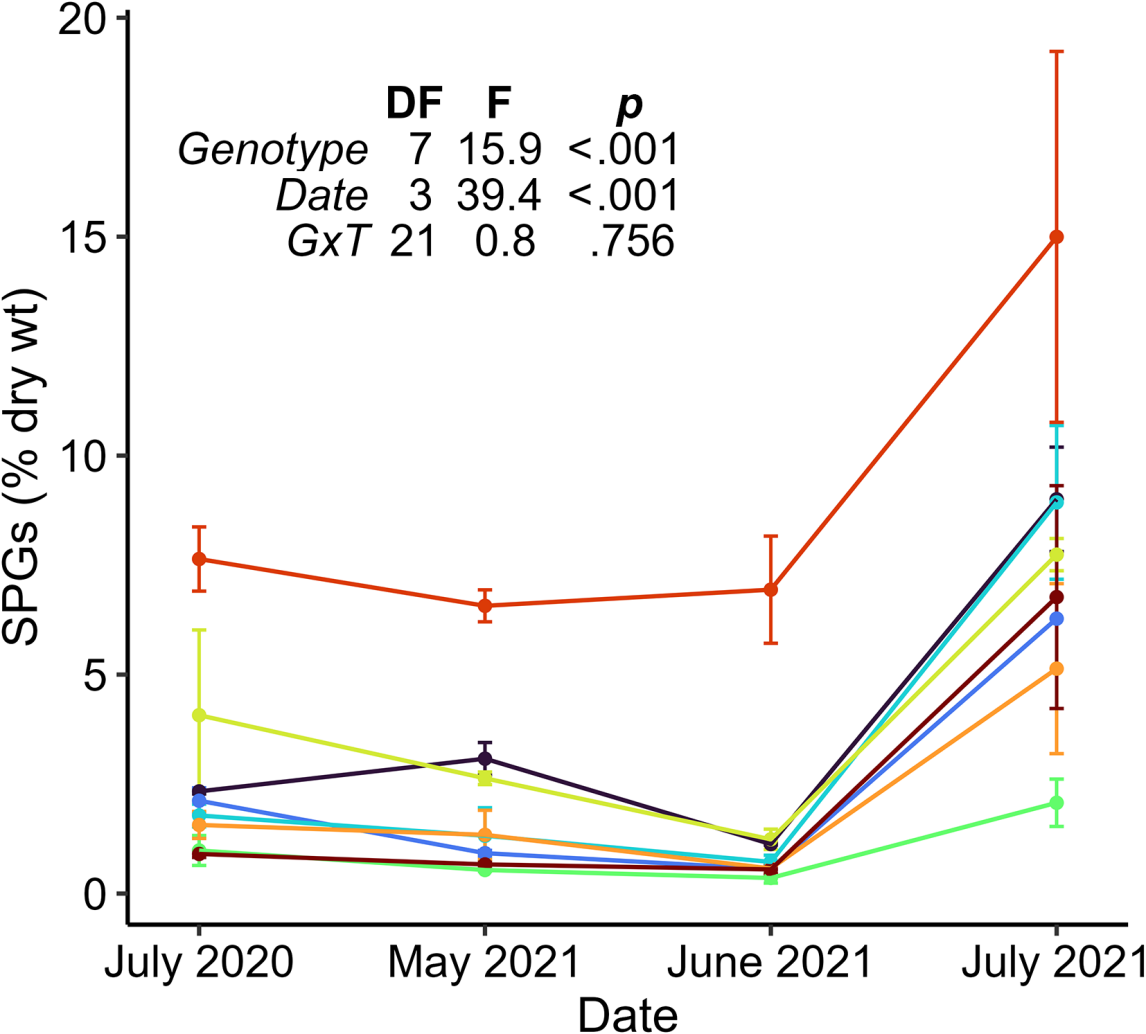
Consequences of spongy moth defoliation for expression of salicinoid phenolic glycosides (SPGs) in an aspen forest of mixed genotypes. Colored lines indicate individual genotypes (8); points and error bars indicate means of total SPG (salicin, salicortin, tremulacin, tremuloidin) concentrations (8 trees per genotype), ±1 standard error. The embedded table shows results from a repeated measures ANOVA, including degrees of freedom, F‐statistics, and *p*‐values for the effects of genotype, time, and their interaction ($R_{\mathrm{adj}}^{2}=.75$). Additional pairwise *t*‐tests among time points revealed that SPG concentrations did not differ among the first three time points (p>.2), but those points differed from the fourth, post‐defoliation time point (p<.001).

We measured levels of SPGs in multiple trees of eight aspen genotypes prior to, during, and after the outbreak. In the year (2020) prior to the outbreak, foliar concentrations of SPGs in the Defoliated forest were comparable to, or moderately lower than, levels in the Undefoliated forest (Figure 6). Immediately preceding and during the defoliation event (May–June 2021), SPG levels in the Defoliated forest varied considerably among genotypes, but not over time (Figure 7). Thereafter, however, SPG levels spiked dramatically in reflushed foliage (July 2021), with an average increase of 8.4‐fold across aspen genotypes. These increases were not simply a consequence of seasonal change. SPG levels vary minimally in mature aspen foliage across a growing season (Falk et al., 2018) and in this case were much higher than in midsummer of the preceding year (July 2020, Figure 7).

### 3.2 Refoliated aspen trees reduce fitness of polyphemus silk moths

**FIGURE 8:**
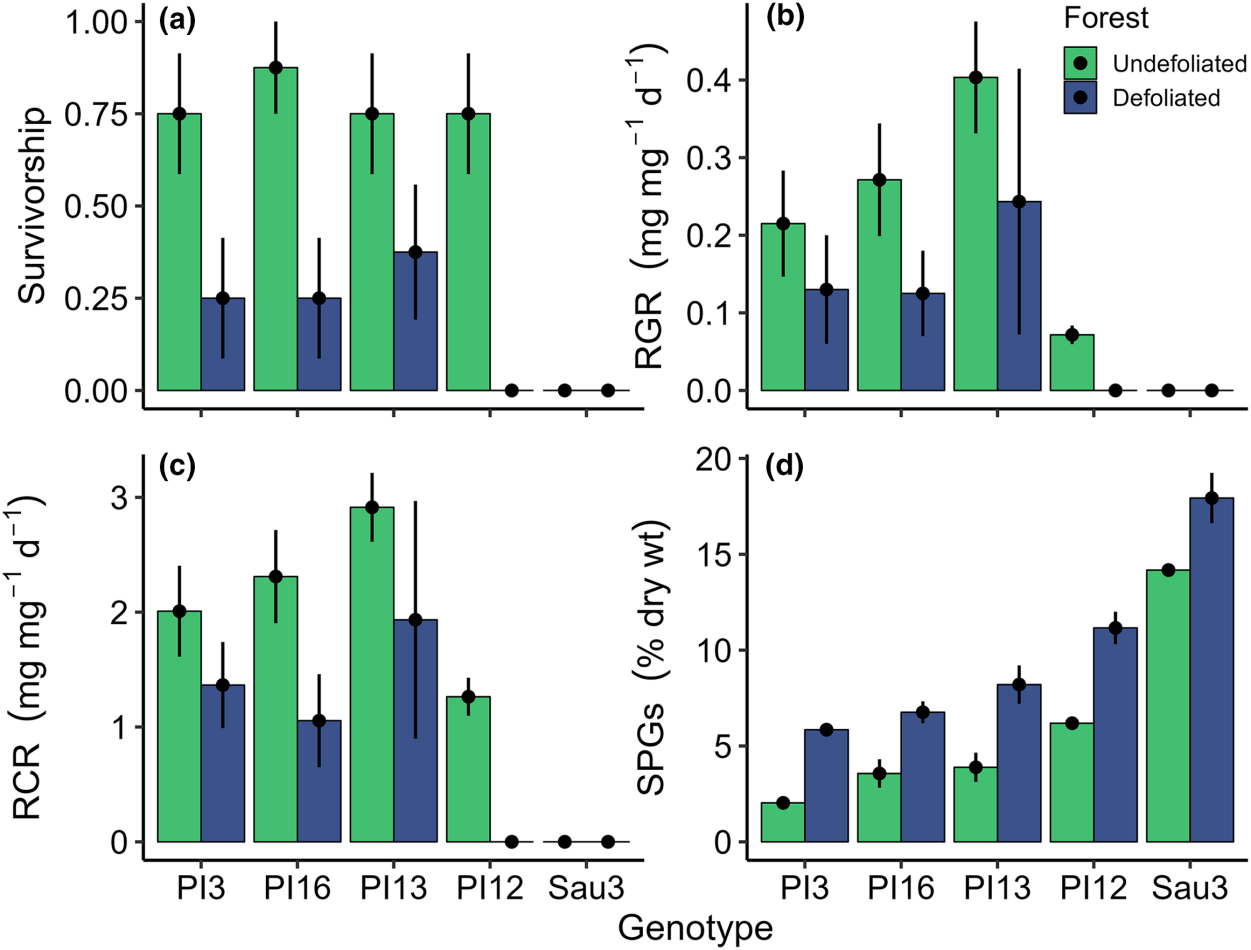
Consequences of spongy moth defoliation for polyphemus larval performance. (a) Survivorship rate. (b) Relative growth rate. (c) Relative consumption rate. (d) Levels of total salicinoid phenolic glycosides in bioassay foliage. Bars represent means (8 replicates, ± 1 standard error) for Undefoliated and Defoliated treatments for each of five aspen genotypes. Corresponding statistics are provided in Table 2.

Survivorship of polyphemus caterpillars declined precipitously when fed reflushed aspen foliage (Defoliated forest), to an average of only 17.5% across aspen genotypes (Figure 8a; Table 1). In contrast, survivorship was high for larvae fed undamaged foliage (Undefoliated forest), with the exception of larvae reared on the single genotype (Sau3) with naturally high (constitutive) levels of SPGs. Of the larvae that survived, those fed leaves from the Defoliated forest had much lower rates of growth and food consumption than did control larvae (Figure 8b,c; Table 2). Levels of SPGs were markedly higher in reflushed, vs. undamaged, bioassay foliage and also varied among aspen genotypes (Figure 8d; Table 2).

**TABLE 1:** Analyses of deviance for survivorship of *Polyphemus* larvae.

Model	R^2^	Source	DF	Dev	Resid DF	Resid dev	*p*
Surv	.52	Null			79	107.68	
		Forest	1	17.66	78	90.02	<.001
		Genotype	4	25.01	74	65.01	<.001
		Forest × Genotype	4	3.41	70	61.6	.492
		Replicate(Genotype)	5	3.84	65	57.76	.572
		Forest × Replicate(Genotype)	5	5.58	60	52.18	.349
Surv (+ SPG)	.69	Null			79	107.68	
		SPGs	1	41.84	78	65.84	<.001
		Forest	1	3.79	77	62.06	.052
		Genotype	4	3.28	73	58.78	.512
		SPGs × Forest	1	1	72	57.77	.317
		SPGs × Genotype	4	13.52	68	44.25	.009
		Forest × Genotype	4	2.21	64	42.04	.697
		Replicate(Genotype)	5	2.67	59	39.36	.750
		SPGs × Replicate (Genotype)	5	6.09	54	33.27	.297
		SPGs × Forest × Genotype	4	0	50	33.27	1.000
		Forest × Replicate (Genotype)	5	0	45	33.27	1.000
		SPGs × Forest × Replicate(Genotype)	5	0	40	33.27	1.000

*Note*: Columns represent model name, McFadden *R* ^2^ statistic, source of variation, degrees of freedom, deviance, residual degrees of freedom, residual deviance, and *p‐*value. For each model, rows represent the additional variation explained by adding the source variable compared with the cumulative variation explained by all preceding variables (reducing the deviance), starting with no factors (Null). Model name indicates whether SPG concentrations were included as an explanatory variable in the model (+ SPG). The underlying fitted models are generalized linear regression models with a binomial link function. A nested tree effect, Replicate(Genotype), is included to account for non‐independence of observations derived from the same tree.

**TABLE 2:** Analyses of variance (ANOVAs) for growth and consumption rates of *Polyphemus* larvae and SPG concentrations of experimental trees.

Model	R^2^_adj_	Source	DF	SS	MS	F	*p*
RGR	.57	Forest	1	0.23	0.23	15.94	<.001
		Genotype	4	0.83	0.21	14.55	<.001
		Forest × Genotype	4	0.05	0.01	0.79	.537
		Replicate(Genotype)	5	0.13	0.03	1.82	.133
		Forest × Replicate(Genotype)	5	0.07	0.01	1.05	.403
		Residuals	36	0.51	0.01		
RGR (+ SPG)	.78	SPGs	1	0.81	0.81	113.38	<.001
		Forest	1	0	0	0.05	.831
		Genotype	4	0.32	0.08	11.22	<.001
		SPGs × Forest	1	0.06	0.06	8.18	.009
		SPGs × Genotype	4	0.15	0.04	5.13	.004
		Forest × Genotype	4	0.13	0.03	4.54	.008
		Replicate(Genotype)	5	0.09	0.02	2.45	.066
		SPGs × Replicate(Genotype)	5	0.03	0.01	0.96	.461
		SPGs × Forest × Genotype	4	0.07	0.02	2.28	.093
		Forest × Replicate(Genotype)	2	0	0	0.02	.982
		SPGs × Forest × Replicate(Genotype)	2	0	0	0.26	.771
		Residuals	22	0.16	0.01		
RCR	.73	Forest	1	17.99	17.99	42.02	<.001
		Genotype	4	44.6	11.15	26.05	<.001
		Forest × Genotype	4	3.67	0.92	2.14	.095
		Replicate(Genotype)	5	4.18	0.84	1.95	.109
		Forest × Replicate(Genotype)	5	2.57	0.51	1.2	.329
		Residuals	36	15.41	0.43		
RCR (+ SPG)	.90	SPGs	1	55.26	55.26	344.64	<.001
		Forest	1	0.26	0.26	1.63	.214
		Genotype	4	10.22	2.55	15.93	<.001
		SPGs × Forest	1	3.35	3.35	20.9	<.001
		SPGs × Genotype	4	3.67	0.92	5.73	.003
		Forest × Genotype	4	6.74	1.68	10.5	<.001
		Replicate(Genotype)	5	2.66	0.53	3.32	.022
		SPGs × Replicate(Genotype)	5	1.12	0.22	1.39	.266
		SPGs × Forest × Genotype	4	1.54	0.39	2.4	.081
		Forest × Replicate(Genotype)	2	0.02	0.01	0.05	.95
		SPGs × Forest × Replicate(Genotype)	2	0.07	0.03	0.2	.817
		Residuals	22	3.53	0.16		
SPG	.91	Forest	1	321.89	321.89	139.69	<.001
		Genotype	4	1498.93	374.73	162.62	<.001
		Forest × Genotype	4	7.14	1.78	0.77	.546
		Replicate(Genotype)	5	73.35	14.67	6.37	<.001
		Forest × Replicate(Genotype)	5	69.81	13.96	6.06	<.001
		Residuals	60	138.26	2.3		

*Note*: Columns represent model name, adjusted *R* ^2^ statistic, source of variation, degrees of freedom, Type I sum of squares, mean squares, two‐tailed *F*‐statistic, and *p*‐value. Model name indicates the response variable (RGR = relative growth rate; RCR = relative consumption rate) and whether SPGs were included as an independent variable (+ SPG) in the model. The underlying fitted models are linear regressions with nested tree effects, Replicate(Genotype), to account for non‐independence of observations derived from the same tree.

### 3.3 Salicinoid phenolic glycosides determine polyphemus performance

**FIGURE 9:**
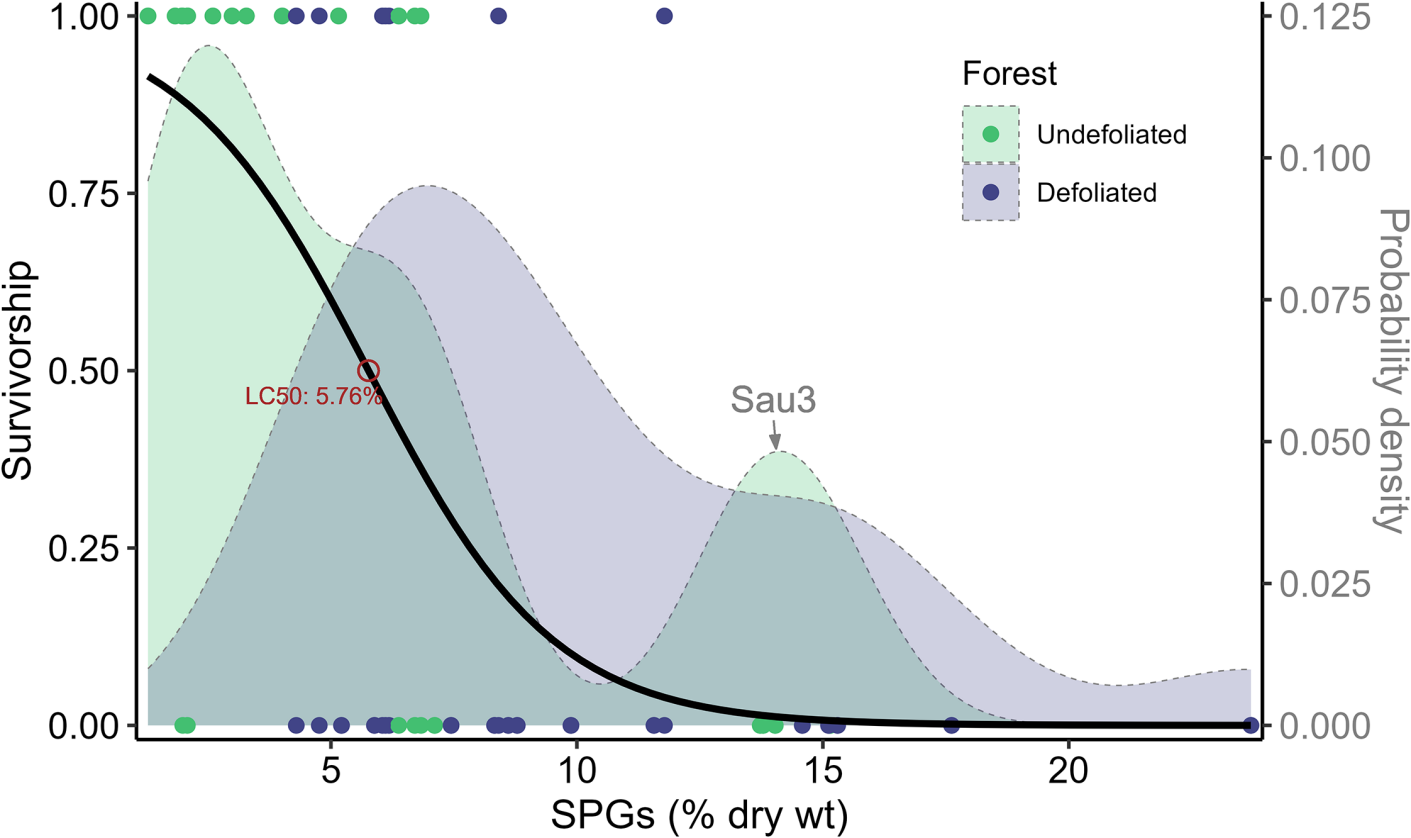
Polyphemus survivorship in relation to foliar salicinoid phenolic glycoside concentration. Points represent individual larval survivorship; the black line represents a logistic regression of larval survivorship (left Y axis) as a function of total SPG concentration (X axis); LC_50_ represents the SPG concentration at which half of the larvae survive. Overlaid curves represent the distribution of SPG concentrations (right Y axis) among trees in the Undefoliated and Defoliated forests that were used for the feeding bioassays. Sau3 indicates an outlier genotype with high constitutive levels of SPGs in the Undefoliated Forest. Tukey's *t*‐tests revealed that the Defoliated forest had average SPG concentrations higher than the Undefoliated forest (p<.001), and the Defoliated forest had average SPG concentrations greater than the LC_50_ (p<.001) while the Undefoliated forest did not (p=.974).

Analysis of deviance for the logistic regression model (Figure 9) showed that variation in larval survivorship was associated with variation in foliar SPG concentrations per se; when that factor was removed, neither aspen genotype nor forest site explained significant variation in insect survivorship.

We then explored the degree to which SPG concentrations contributed to the observed differences in polyphemus performance. Across all treatments, the LC_50_ (lethal concentration for 50% of polyphemus larvae) of SPGs was 5.8% leaf dry weight (Figure 9). With the exception of one genotype (Sau3), most trees in the Undefoliated forest had SPG concentrations well below that level. Defoliation, however, shifted the distribution of SPG concentrations, markedly increasing the toxicity of the aspen phytochemical landscape (Figure 9). Comparison of polyphemus survivorship models, with and without SPG terms (Table 1), revealed that differences in larval mortality between forests and among genotypes were entirely explained by SPG concentration. Similarly, forest effects on polyphemus growth and consumption rates were entirely attributable to SPGs, although genotype had additional effects beyond SPG concentrations alone (Table 2).

## 4 DISCUSSION

In this study, we demonstrate that an invasive forest insect species can strongly reduce the fitness of a charismatic native species by a heretofore undocumented mechanism: a multifold increase in toxicity of the phytochemical landscape. Spongy moths completely defoliated a forest of mixed aspen genotypes in early summer. The trees responded by producing a second flush of leaves with exceptionally high levels of salicinoid phenolic glycosides. Those concentrations, in turn, were toxic to native polyphemus caterpillars that typically feed on aspen in mid‐summer.

Factors contributing to variation in foliar SPG levels have been broadly investigated for trembling aspen in North America (Barker, Holeski, & Lindroth, 2019; Lindroth & St. Clair, 2013). For mature trees, levels vary highly among genotypes, but minimally in response to environmental factors (e.g., light and nutrient availability, leaf damage), canopy location, and leaf age after expansion (Barker, Holeski, & Lindroth, 2019; Cope et al., 2019; Donaldson et al., 2006; Eisenring et al., 2021; Falk et al., 2018; Lindroth et al., 2023; Lindroth & St. Clair, 2013). When defoliation from insects is sufficient to elicit a second leaf flush, however, levels of SPGs increase greatly. Donaldson and Lindroth (2008) previously monitored SPG levels in six aspen genotypes during and after a forest tent caterpillar (*Malacosoma disstria*) outbreak in northern Wisconsin and described patterns that mirror those reported here.

Aspen is a primary food source for well over 100 species of insects and mammals, but even aspen‐adapted species suffer reductions in fitness when levels of SPGs are high. Spongy moths, forest tent caterpillars, Canadian tiger swallowtails (*Papilio canadensis*), big poplar sphinx moths (*Pachysphinx modesta*), and white‐marked tussock moths (*Orgyia leucostigma*) feed heavily or exclusively on aspen, but exhibit reduced feeding, growth, and survivorship on high‐SPG trees (Agrell et al., 2000; Hemming & Lindroth, 1995; Hwang & Lindroth, 1998; Lindroth & St. Clair, 2013). Mammalian browsers such as elk (*Cervus canadensis*) and porcupine (*Erethizon dorsatum*) also feed extensively on aspen, but are deterred by high SPG concentrations (Diner et al., 2009; Wooley et al., 2008). In short, the chemical consequences of spongy moth defoliation likely reverberate throughout the diverse, native aspen‐feeding community. For example, Redman and Scriber (2000) reported that growth and survivorship of the northern tiger swallowtail (*Papilio canadensis*) declined on aspen foliage damaged by spongy moths (but did not assess defense chemistry). Moreover, phytochemically‐mediated indirect effects of spongy moth defoliation may occur over expansive areas. In recent decades, spongy moths have become established throughout most of the Great Lakes Region, where aspen is a dominant tree species. In Michigan alone, aspen forest type occupies nearly a million hectares, and spongy moths defoliated over 500,000 ha of Michigan forest in 2021 (Michigan Department of Natural Resources, 2022).

Spongy moth‐elicited increases in the toxicity of the phytochemical landscape have potential knock‐on effects for ecological structure and function well beyond the fitness of individual species of herbivores. Aspen is a foundation species (Ellison et al., 2005), and many of its diverse ecological interactions are phytochemically governed (Lindroth & St. Clair, 2013). Aspen forests are recognized as biodiversity hotspots for insects, birds, and mammals (Martin & Maron, 2012; Rogers, 2017). The richness and abundance of aspen canopy insects is linked to foliar chemistry (Barker et al., 2018; Morrow, 2022), and multifold increases in defense expression are likely to reduce the diversity of those communities. Multi‐trophic interactions may also be disrupted, as SPGs can affect the behavior and fitness of natural enemies (birds, parasitoids) of aspen‐feeding insects (Müller et al., 2006; Roth et al., 1997). Finally, forest carbon dynamics may be compromised by the chemical legacy effects of spongy moth defoliation. Trembling aspen is a major driver of carbon flux and sequestration in boreal forests of North America (Laganière et al., 2015). Carbon fixation is reduced, of course, by massive losses of photosynthetic tissue and recruitment of stored carbon reserves for leaf reflush. Our work, however, suggests that forest carbon sequestration may also be diminished by a secondary, under‐recognized mechanism: the reallocation of fixed carbon from growth to defense. Synthesis of SPGs exacts a significant cost to tree growth (Kruger et al., 2020). Consequently, sizeable increases in expression of SPGs across an aspen forest landscape likely reduce the potential of that landscape to sequester carbon in the form of tree biomass.

Forest defoliation by invasive species such as spongy moths can influence the abundance and diversity of co‐occurring insect species via a cascade of diverse direct and indirect effects (Gandhi & Herms, 2010; Kenis et al., 2009). Defoliation changes the microclimate of forest canopies, and shifts in microclimate can alter insect communities (Sallé et al., 2021). Defoliation reduces food availability, which may negatively affect other herbivorous species via increased competition (Kenis et al., 2009; Work & McCullough, 2000). Outbreaks of defoliating insects can also modify the magnitude of top‐down impacts on other insect species. For example, Redman and Scriber (2000) reported elevated rates of parasitism on northern tiger swallowtail caterpillars near spongy moth infestations. Clearly, numerous direct and indirect community‐level interactions mediate the effects of defoliating insects on other members of the arthropod community. Our work shows that an additional mechanism – increases in toxicity of the phytochemical landscape – should be incorporated into that panoply of factors.

Biodiversity conservation work has historically prioritized protection of rare species. Recently, however, the need for protecting against the loss of common species on which many other species depend (i.e., foundation species such as aspen), has been emphasized (Austin & Ballaré, 2023). Austin and Ballaré (2023) assert that elimination of such species as a consequence of ecological interactions may fundamentally alter biodiversity and ecosystem function. The results from our work suggest that in addition to quantitative loss, wholesale qualitative change of foundation species may have similar consequences.

“Reducing threats to biodiversity” is the first of 23 global targets for urgent action articulated in the 2022 Kunming‐Montreal Global Biodiversity Framework (https://www.cbd.int/gbf/targets/). Progress toward that goal will require a broad range of research into the scope, magnitude, and context‐specificity of the diverse drivers of biodiversity loss. Heretofore, assessments of the impacts of invasive insects on native insects have focused on direct competition, changes in plant community composition, disruption of food web dynamics, and collateral damage from control strategies (e.g., insecticides) (Gandhi & Herms, 2010; Kenis et al., 2009). To those should be added investigation of potential indirect, phytochemically‐mediated consequences of invasive insect herbivory, which may exert long‐term and widespread legacy effects on native species, community diversity, and ecosystem function.

## AUTHOR CONTRIBUTIONS

**Richard L. Lindroth:** Conceptualization (lead); funding acquisition (lead); investigation (supporting); methodology (lead); project administration (equal); resources (lead); supervision (supporting); writing – original draft (lead); writing – review and editing (lead). **Mark R. Zierden:** Investigation (equal); methodology (supporting); writing – review and editing (supporting). **Clay J. Morrow:** Data curation (equal); formal analysis (lead); writing – review and editing (supporting). **Patricia C. Fernandez:** Conceptualization (lead); data curation (supporting); formal analysis (supporting); funding acquisition (supporting); investigation (lead); methodology (equal); project administration (equal); supervision (equal); writing – review and editing (supporting).

## CONFLICT OF INTEREST STATEMENT

None of the authors has any competing interest, financial or otherwise, with the research reported in this paper.

## Data Availability

The data and supporting code are available at the Dryad repository, doi:10.5061/dryad.41ns1rnks.
